# Evaluation of iStent Inject® Trabecular Microbypass Stent Implantation With Phacoemulsification in Cases of Mild to Moderate Primary Open-Angle Glaucoma (POAG)

**DOI:** 10.7759/cureus.96530

**Published:** 2025-11-10

**Authors:** Alhumam Alkhusheh, Rosina Zakri, Mohamed Abdelhady

**Affiliations:** 1 Kent, Surrey and Sussex Foundation School, Maidstone and Tunbridge Wells NHS Trust, Kent, GBR; 2 Ophthalmology Department, East Kent Hospitals University NHS Foundation Trust, Canterbury, GBR; 3 Ophthalmology Department, Maidstone and Tunbridge Wells NHS Trust, Kent, GBR

**Keywords:** iop monitoring, istent inject, medication burden, phacoemulsification cataract surgery, primary open-angle glaucoma

## Abstract

Background: The iStent inject® (second generation) is an example of a minimally invasive glaucoma surgery (MIGS) device being increasingly used in the management of glaucoma. It is a recent advancement in the field of glaucoma treatment and can be performed simultaneously with cataract surgery.

Objectives: The primary objective is to evaluate the change in intraocular pressure (IOP), with secondary objectives to assess the medication burden and safety profile of the implantation of the two second-generation iStent inject trabecular microbypass stents with concomitant phacoemulsification.

Methods: A service evaluation of the procedure was conducted over multiple hospital sites across the East Kent Hospitals University NHS Foundation Trust. A data set of 102 eyes was generated, which underwent combined phacoemulsification and iStent inject implantation under three iStent surgeons. This data set included information on preoperative and postoperative IOP, complications, and the number of glaucoma medications established.

Results: There was a reduction of 43.5% in mean IOP in the 102 eyes from a preoperative IOP mean of 22.0 ± 8.54 mmHg (mean ± standard deviation) to a postoperative IOP of 12.4 ± 4.21 mmHg (p < 0.001). There was a reduction in the mean number of medications in the overall cohort by 11.7% from 2.74 ± 1.15 preoperatively to 2.42 ± 1.01 postoperatively (p < 0.001). Ninety (88.2%) eyes had a reduction of ≥20% in IOP. No intraoperative complications were recorded, and two (2%) eyes developed cystoid macular oedema (CMO) postoperatively. There were no other adverse effects or complications reported.

Conclusions: Significant reductions in IOP and medication burden were identified in this cohort of 102 eyes. It has been observed to be a safe surgery with a high safety profile. The utilization of this concomitant surgery could help reduce the number of follow-ups required for patients with glaucoma compared to exclusively medical control or advanced glaucoma surgery and lead to significantly less theater time.

## Introduction

Glaucoma is a general term that encompasses a group of diseases characterized by progressive degeneration of the optic nerve with subsequent visual field loss. It is the leading cause of irreversible blindness worldwide, affecting more than 70 million patients, many of whom are known to have the diagnosis. Glaucoma itself can manifest in a variety of ways, but in the majority of cases, patients succumb to a painless loss of vision until the late stages of the disease [1]. This has been reiterated in a series of published literature, including the systematic review by Soh et al. (2021), which demonstrated that over 50% of all glaucoma cases were previously undetected, regardless of geographical location [2].

There are numerous risk factors for the development of glaucoma, including age, family history, and previous eye trauma/operations, but the most important of these is intraocular pressure (IOP), which remains the only clinically controllable variable [3]. Subsequently, IOP control in patients with glaucoma is essential and forms the mainstay of treatment. There are many published studies and trials investigating the risk of progression and IOP control: the Early Manifest Glaucoma Trial, which estimated a 10% lower risk of glaucoma progression for each 1 mmHg reduction in IOP [4], and alternatively, the Advanced Glaucoma Intervention Study (AGIS), which demonstrated that eyes with an average IOP greater than 17.5 mmHg had an estimated worsening during subsequent follow-up with one unit of visual field defect score greater detected than in eyes with an average intraocular pressure less than 14 mmHg [5].

Over the last few decades, there have been huge advancements in treatment approaches to glaucoma. These include medical interventions (largely made up of topical medications), injections, laser therapies, and surgical interventions.

The use of topical ophthalmic medications remains the most preferred route of drug administration in diseases affecting the anterior segment, due to their non-invasive and simple application [6]. However, due to the detailed anatomy of the eye, the topical administration bioavailability is low. Alternative factors that impact this range from the limited absorptive surface of the cornea, to the tear film turnover, to reflex blinking, to patient compliance [7]. Due to such factors, less than 5% of drops reach deeper ocular tissue and do not penetrate the posterior segment of the eye adequately to reach therapeutic drug concentrations. Hence, medications targeting the posterior segment include different modes of administration, such as intravitreal injections or systemic administration [6].

Patient compliance is a very important factor affecting the efficacy of topical medication. There are many concerns raised by patients regarding drop use, the majority of these around declining cognitive impairment and failing grip strength [8]. Alternative therapies include selective laser trabeculoplasty (SLT), which is growing in popularity, with organizations such as the UK’s National Institute for Health and Care Excellence (NICE) recommending it as a first-line treatment in cases of newly diagnosed chronic open-angle glaucoma (COAG) or ocular hypertension (OHT) [9]. This, too, however, has associated adverse effects and an unpredictable efficacy profile, with some studies demonstrating a temporary effect, largely lasting around 3-5 years after a single treatment [10].

Regardless of medical intervention, there remain cases where surgical intervention is indicated due to the uncontrolled nature of the disease. Historically, what was previously largely limited to trabeculectomies has seen a dramatic change in the possibilities of procedures in the last two decades [11]. This has included the recent introduction of minimally invasive glaucoma surgery (MIGS), minimally invasive surgeries aimed at reducing IOP and reducing the dependence on topical ophthalmic medications.

An example of a MIGS device is the iStent inject® (second generation). This is a device that can be implanted simultaneously during cataract surgery, using the same incision, and aims to bypass the trabecular meshwork to allow the direct passage of aqueous humor into Schlemm’s canal. Published studies demonstrate that the iStent inject offers significant reductions in IOP and dependence on drop medications with a relatively low long-term risk [11,12].

This service review hopes to demonstrate the effectiveness and safety of implanting iStent inject® second-generation trabecular microbypass stents in conjunction with phacoemulsification in patients with mild to moderate primary open-angle glaucoma (POAG) and cataracts. The primary objective was to evaluate reductions in IOP postoperatively, with secondary objectives including evaluation of the side effect profile and medication burden.

This article has been accepted to be presented as a poster at the 15th Low Vision Research and Rehabilitation Congress on September 8-12, 2025.

## Materials and methods

Study design

A service evaluation was undertaken to investigate the efficacy of implanting the iStent inject in conjunction with cataract surgery in the department. A data set of 107 eyes (98 patients) was initially generated. These were patients who had undergone a combined cataract surgery and iStent inject from February 2022 to December 2023 over multiple sites across the same trust (East Kent Hospitals University NHS Foundation Trust). These included surgeries performed and approved by three iStent surgeons.

Baseline demographics and ocular characteristics of each patient were obtained to include age, sex, eye, preoperative medicated IOP, and the number of preoperative topical ophthalmic medications.

The patients were then requested to be seen in the clinic post-procedure; this ranged from two weeks to three months after surgery. Information was then further gathered to include postoperative complications, adverse effects, postoperative medicated IOP, and the number of topical ophthalmic medications used.

Four patients (five eyes) did not attend any follow-up appointments post-procedure and were hence excluded from the data set prior to data analysis. As such, results and conclusions were drawn from the remaining 102 eyes (94 patients).

Inclusion and exclusion criteria

The inclusion criteria consisted of all patients operated with a combined phacoemulsification and iStent inject procedure over the specified time frame, patients of all ages and gender, patients with previous glaucoma procedures, patients of all ethnicities, patients of all stages of glaucoma that are eligible for the iStent procedure (under NICE guidance), and patients including one or both eyes, where each eye was recorded as a single data set.

The exclusion criteria included patients with multiple comorbidities, patients who underwent the iStent procedure alone but no cataract surgery, patients with previous glaucoma surgeries, patients who did not consent or were unable to consent to their data being used for any reason, and patients who had the procedure combined or followed by any other procedure within the given time frame.

Main outcome measures

Primary outcome measures included IOP evaluation using preoperative and postoperative IOP. The cohort was stratified into three groups dependent on baseline (preoperative) IOP (IOP ≤ 15 mmHg, 15 < IOP ≤ 21 mmHg, and IOP > 21 mmHg), in addition to patients who experienced a ≥20% IOP decrease from baseline at the follow-up visits.

Secondary outcomes included evaluating the medication burden and safety profile. The number of glaucoma medications established preoperatively and postoperatively was analyzed to investigate changes in medication burden.

There were eight patients who contributed information on both eyes to the study; subsequently, each eye was considered an independent unit of observation for the purposes of the study. Patients with ophthalmic comorbidities were excluded from the study.

To aid in presenting preoperative and postoperative data, descriptive statistics were used. Preoperative IOP was compared to postoperative IOP using the paired two-tailed t-test for dependent samples. A p-value of 0.05 or less was considered statistically significant.

Ethical approval and consent to participate

Registration of the project was not required as the study design is one of a service evaluation; this was emphasized by the local research and development and audit departments of the trust. Patients who were due to have the procedure were seen in the clinic and consented as per routine. Similarly, demographics and IOP were recorded as per the normal standard of care. They were then consented for the data to be used as part of the study. As this was an observational study, they did not need a cool-off time to consider for any great length of time; however, they were given the opportunity to withdraw from the study if they wished to. They were given a leaflet for further information, and the protocol was in line with normal standards of care and information recording. No additional information/randomization, or appointment was sought for the purpose of the study.

Data was stored as per data security law in password-protected spreadsheets and shared with the audit department. As per protocol, participation was recorded in the notes along with consent.

## Results

Baseline characteristics

A total of 102 eyes completed the operation and attended follow-up in the clinic after the procedure. The mean age for the cohort was 74.9 (standard deviation ± 9.11), with a male predominance. The age ranged between 55 and 99 years old. Table 1 illustrates the baseline demographics and ocular characteristics collected.

**Table 1:** Baseline demographics and ocular characteristics SD: standard deviation, IOP: intraocular pressure

Baseline demographics and ocular characteristics		Total
Number	Patients	94
	Eyes	102
Age (years)	Mean ± SD	74.9 ± 9.11
	Range	55-99
Sex	Male	50
	Female	44
Eye	OD	53
	OS	49
Baseline (preoperative) IOP (mmHg)	Mean ± SD	22.0 ± 8.54
Medications	Mean ± SD	2.74 ± 1.15

Primary objective: IOP evaluation

**Figure 1:**
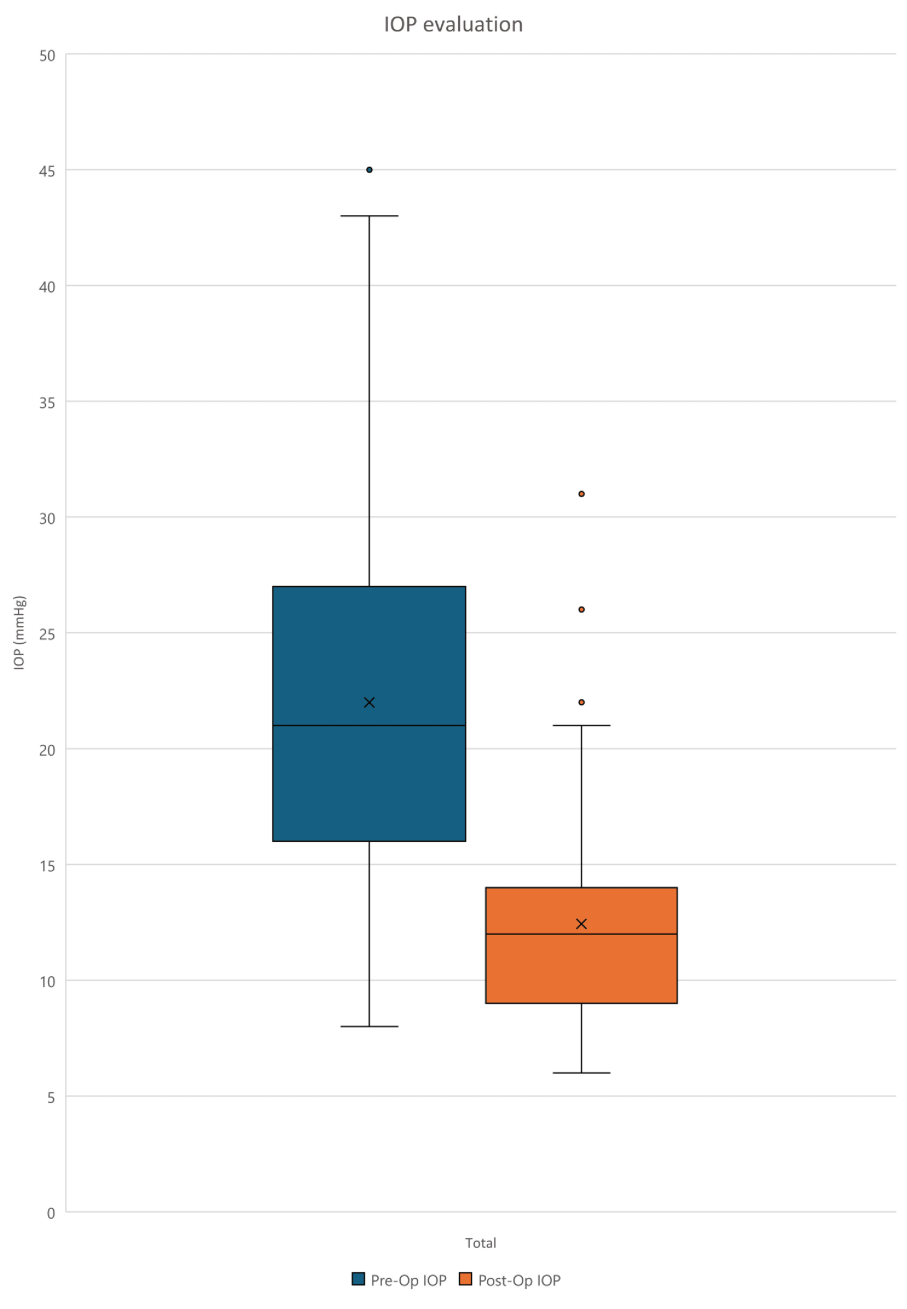
Box and whisker plot comparing preoperative and postoperative IOP The box represents the interquartile range (25th to 75th percentile); the ‘X’ represents the mean; the horizontal line represents the median (50th percentile); the upper and lower vertical lines represent 95th and 5th percentiles, respectively; dots represent outliers. IOP: intraocular pressure

There was a reduction of 43.5% in mean IOP in the 102 eyes from a preoperative IOP mean of 22.0 ± 8.54 mmHg (mean ± standard deviation) to a mean postoperative IOP of 12.4 ± 4.21 mmHg (p < 0.001). Seven (6.9%) eyes were unable to reach an IOP less than 18 mmHg, and no patient had an IOP less than 6 mmHg (Figure 1).

**Figure 2:**
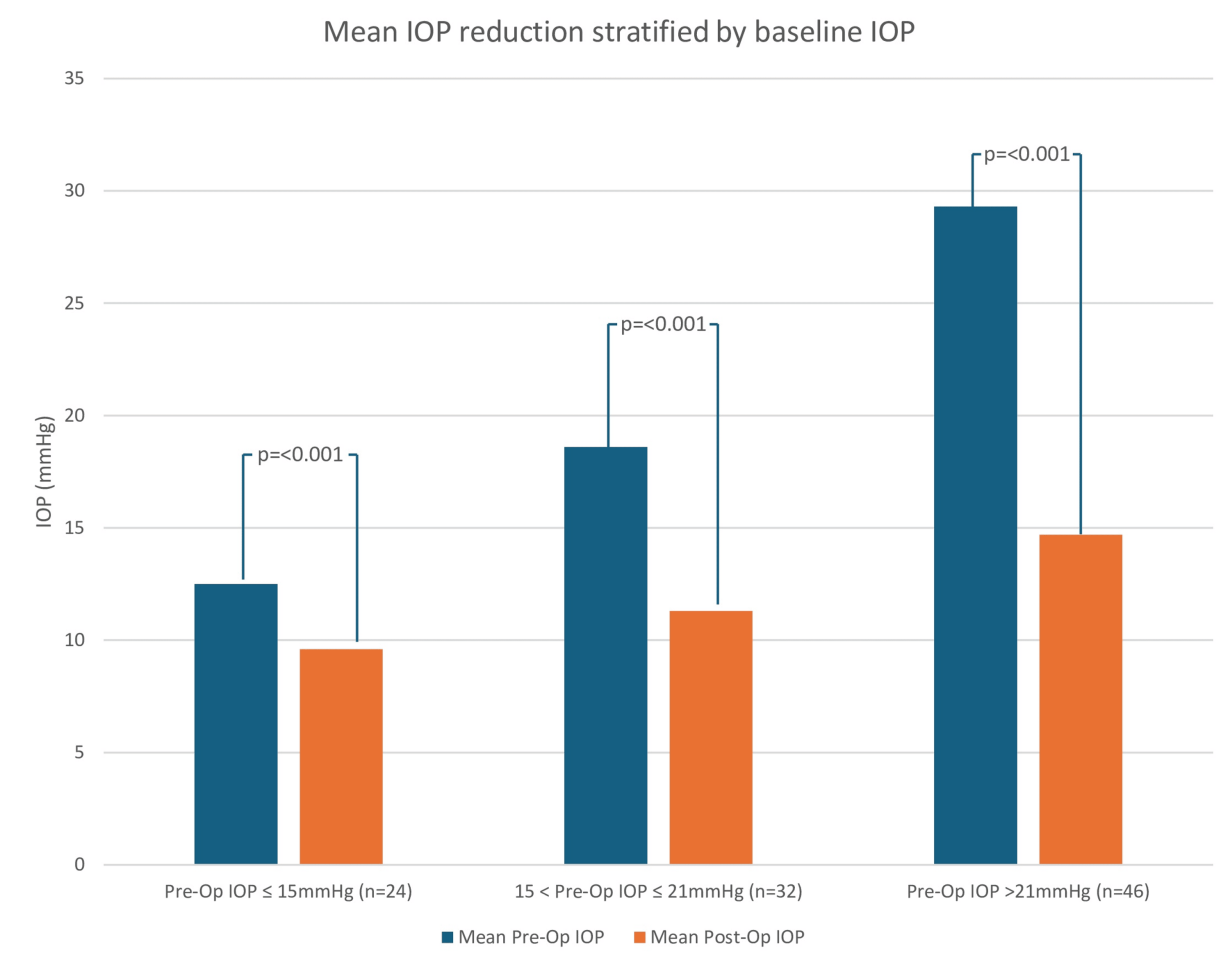
IOP evaluation: bar graph demonstrating the reduction in mean preoperative and postoperative IOP, stratified into three categories based on baseline IOP: ≤15 mmHg (n = 24), 15-21 mmHg (n = 32), and >21 mmHg (n = 46) IOP: intraocular pressure

The data set (n = 102) was further stratified into three groups, based on baseline (preoperative) IOP: ≤15 mmHg (n = 24), 15-21 mmHg (n = 32), and >21 mmHg (n = 46). The mean IOP in each of these groups were compared before and after the procedure (p < 0.001 in all three groups) (Figure 2).

**Figure 3:**
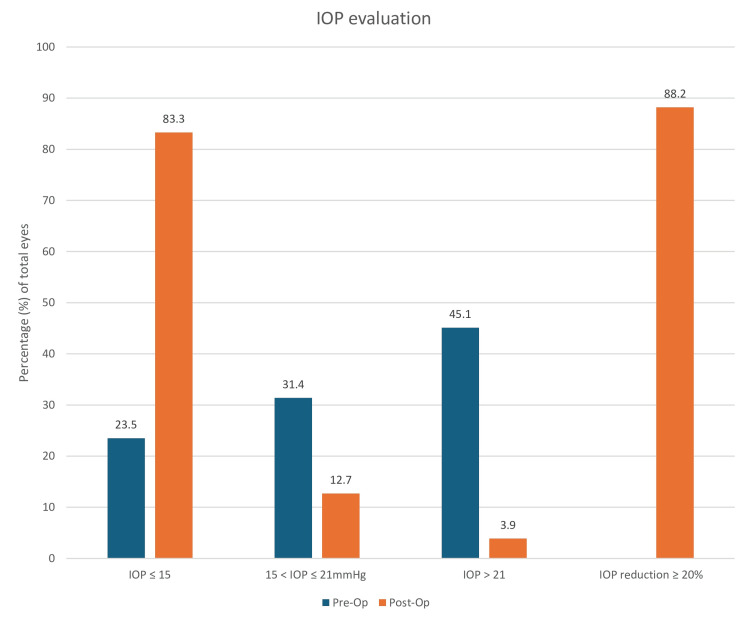
IOP evaluation: graph illustrating the proportion (percentage) of the cohort, both preoperatively and postoperatively, which fall into each of the three categories: IOP ≤ 15 mmHg, 15 < IOP ≤ 21 mmHg, and IOP > 21 mmHg; there is also a final bar to illustrate the percentage of eyes that experienced a reduction of IOP ≥ 20% postoperatively IOP: intraocular pressure

Prior to the operation, 24 (23.5%) eyes made up the group with IOP ≤ 15 mmHg. After which, 85 (83.3%) eyes had a postoperative IOP ≤ 15 mmHg. The proportion changes of the case series for the other two groups (15-21 mmHg and >21 mmHg) are also seen in Figure 3. At the follow-up appointment, 90 (88.2%) eyes showed a reduction of 20% IOP after the procedure (Figure 3).

Secondary objectives: Medication burden and safety profile

There was a reduction in the mean number of medications in the overall cohort by 11.7% from 2.74 ± 1.15 preoperatively to 2.42 ± 1.01 postoperatively (p < 0.001).

**Figure 4:**
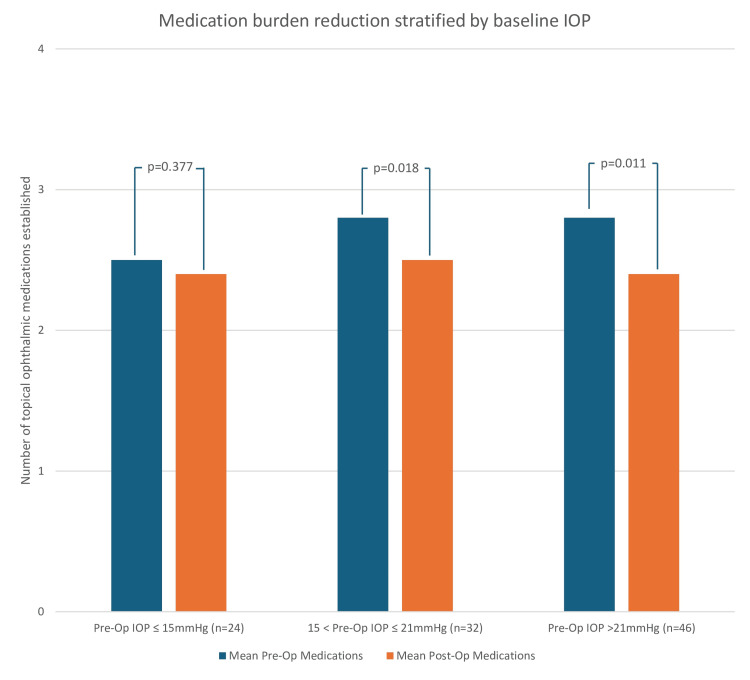
Bar graph demonstrating the change in the mean number of glaucoma medications preoperatively and postoperatively, stratified into three categories based on baseline IOP: ≤15 mmHg (n = 24), 15-21 mmHg (n = 32), and >21 mmHg (n = 46) IOP: intraocular pressure

The data set of 102 eyes was stratified into the same three groups, based on baseline (preoperative) IOP: ≤15 mmHg (n = 24), 15-21 mmHg (n = 32), and >21 mmHg (n = 46). The mean number of medications established in each of these groups were compared before and after the procedure. Statistical significance was identified in the latter two groups (p = 0.018 in the group with preoperative IOP of 15-21 mmHg and p = 0.011 in the group with preoperative IOP > 21 mmHg), with an insignificance noted in the group with preoperative IOP ≤ 15 mmHg (p > 0.3) (Figure 4).

**Figure 5:**
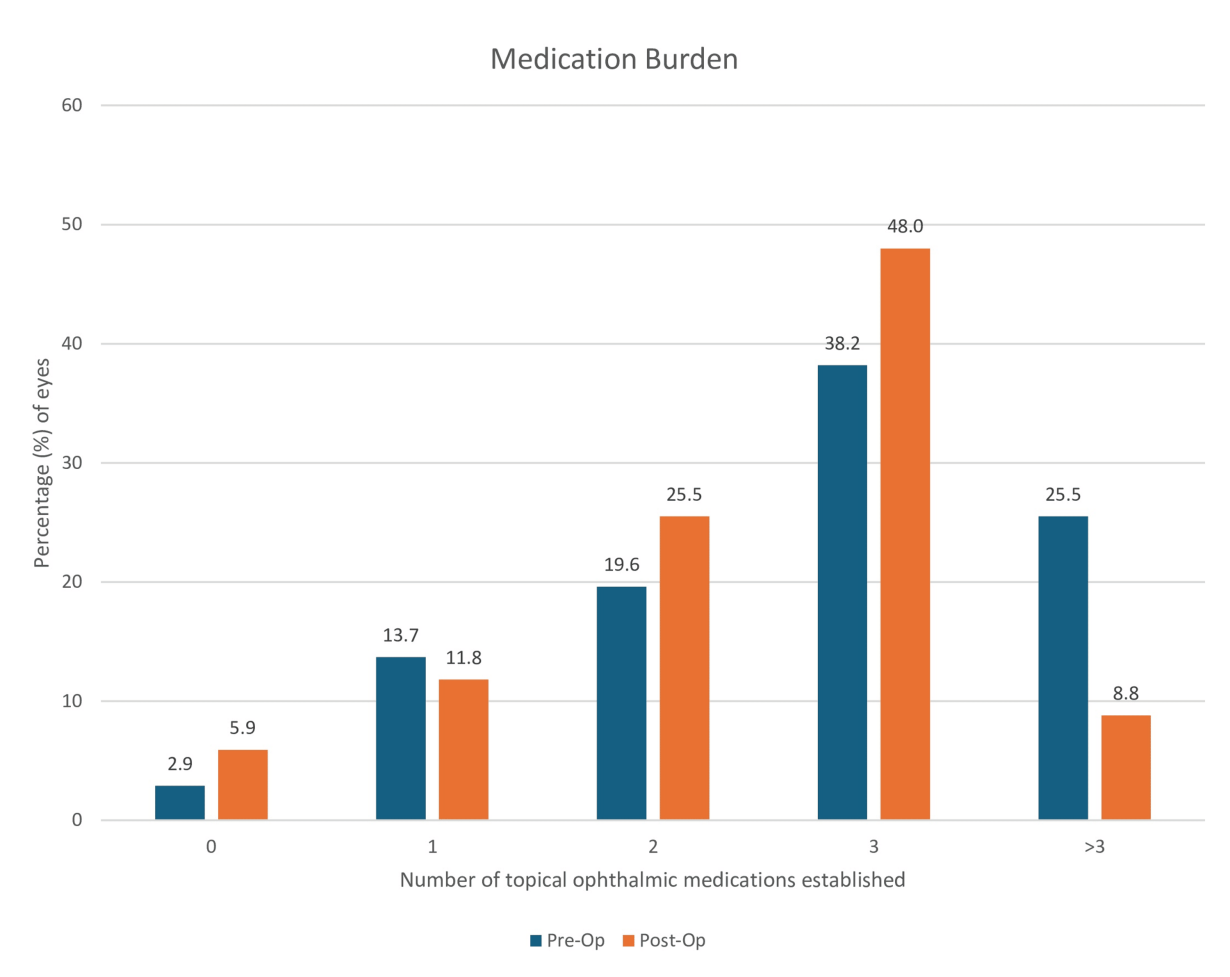
Medication burden: bar graph illustrating the proportion (percentage) of the 102 eyes established on the number of topical ophthalmic medications, comparing preoperatively and postoperatively

The proportion of the number of eyes established on each number of topical ophthalmic medications was also analyzed. For instance, preoperatively, 26 (25.5%) eyes were established on >3 medications; this was then reduced to nine (8.8%) eyes postoperatively. Additionally, there were three (2.9%) eyes established on 0 medications preoperatively, which then increased to six (5.9%) eyes postoperatively. The cohort was categorized into five different groups dependent on the number of medications they were established on: 0, 1, 2, 3, and >3 medications. The remainder of the proportion changes can be observed in Figure 5.

Of the 102 eyes in this cohort that underwent implantation of the iStent inject stents in conjunction with phacoemulsification, there were no intraoperative complications recorded. Two (2%) eyes later developed cystoid macular oedema (CMO). There were no reports of adverse effects such as hypotony, hyphema, or corneal decompensation.

## Discussion

The objective of this service review was to attempt to demonstrate the effectiveness and safety of implanting the iStent inject® second-generation trabecular microbypass stents in conjunction with phacoemulsification in patients with mild to moderate primary open-angle glaucoma (POAG) and cataract. The aim was set to achieve a reduction of at least 20% in IOP post-procedure, which was achieved in 90 (88.2%) eyes.

The box and whisker plot observed in Figure 1 shows a reduction in median IOP after surgery, as indicated by the lower position of the orange box compared to the blue box. The range of IOP values is also narrower after surgery, as shown by the smaller size of the orange box and shorter whiskers, indicating less variability in postoperative IOP compared to preoperative IOP. Overall, the data suggest that the iStent inject procedure was effective in lowering intraocular pressure and in making the results more consistent and predictable.

The follow-up was attended by 94 (95.9%) patients, with four (4.1%) patients not attending. Due to hospital pressures and practicalities, the follow-up period ranged from two weeks to three months.

The concomitant phacoemulsification and implantation of the iStent devices demonstrated a high safety profile with no reports of adverse effects such as hypotony, hyphema, or corneal decompensation.

A statistically significant reduction in mean IOP was demonstrated by 43.5% in patients undergoing a combined phacoemulsification and iStent inject implantation. A similar retrospective study conducted by Neuhann and Neuhann [3] also demonstrated a statistically significant reduction in IOP by 25.5% at 12 months postoperatively and 26.6% at 24 months postoperatively in 164 eyes undergoing this surgical combination, too. However, a difference noted between the two studies is that they reported a markedly large reduction in mean medication burden of 85% at 12 months postoperatively and 81% at 24 months postoperatively. In contrast, this case series reports an 11.7% reduction in the number of glaucoma medications used postoperatively. There are numerous possible explanations for this; for instance, this may be due to the longer follow-up period of 12 and 24 months, in turn, perhaps demonstrating a prolonged therapeutic effect of the combined surgery. Alternatively, perhaps, it is due to the nature of the primary diagnoses, as their study included several subtypes of glaucoma, while this study included mostly that of POAG [3].

Limitations

This service review of the iStent inject does have limitations that should be taken into consideration. For instance, the lack of baseline parameters that may have proven helpful, such as the primary diagnosis or visual acuity, or C/D ratios, to further investigate optic nerve health. While the majority of patients included in this study had a background of mild to moderate POAG, this was not one of the parameters collected at baseline. Additionally, the short range of the follow-up period (two weeks to three months) used is a limitation that could be increased in future studies to aid assessment for a sustained reduction in IOP.

## Conclusions

Significant reductions in IOP and medication burden were identified in this cohort of 102 eyes that underwent a concomitant iStent injection and phacoemulsification surgery, in which the majority of cases were of mild to moderate POAG. It has been observed to be a safe surgery with a high safety profile. The utilization of this concomitant surgery could help reduce the number of follow-ups required for patients with glaucoma compared to exclusive medical control or advanced glaucoma surgery and lead to significantly less theater time.
